# 4-(Thio­phen-2-yl)-2-[4-(tri­fluoro­meth­yl)phen­yl]-2,3-di­hydro-1,5-benzo­thia­zepine

**DOI:** 10.1107/S1600536814002529

**Published:** 2014-02-08

**Authors:** B. C. Manjunath, M. Manjula, K. R. Raghavendra, K. Ajay Kumar, N. K. Lokanath

**Affiliations:** a Department of Studies in Physics, Manasagangotri, University of Mysore, Mysore 570 006, India; b Department of Studies in Chemistry, Manasagangotri, University of Mysore, Mysore 570 006, India; c Post Graduate Department of Chemistry, Yuvaraja’s College, University of Mysore, Mysore 570 006, India

## Abstract

In the title compound, C_20_H_14_F_3_NS_2_, the seven-membered thia­zepine ring adopts a slightly distorted twist–boat conformation. The mean plane of the five-membered thio­phene ring fused to the thia­zepine ring is twisted by 32.3 (3) and 55.6 (4)° from the benzene and phenyl rings, respectively. In the crystal, inversion dimers linked by pairs of weak C—H⋯N inter­actions are observed.

## Related literature   

For the biological activity of 1, 4-thia­zepines, see: Skiles *et al.* (1986 ▶); Zeng & Alper (2010 ▶). For a related structure, see: Manjula *et al.* (2013 ▶). For standard bond lengths, see: Allen *et al.* (1987 ▶). 
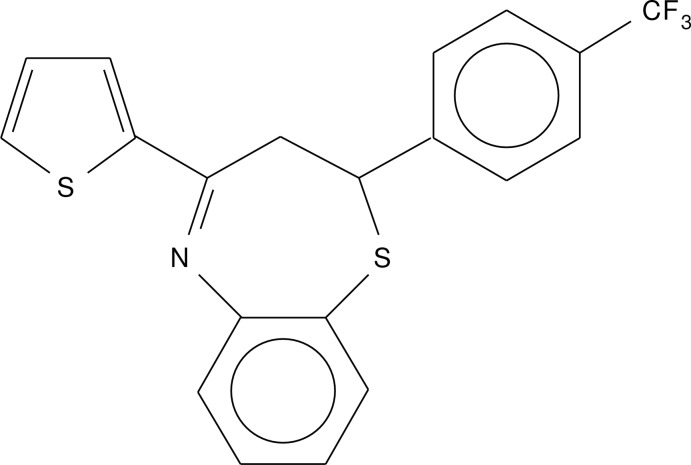


## Experimental   

### 

#### Crystal data  

C_20_H_14_F_3_NS_2_
*M* *_r_* = 389.46
Orthorhombic, 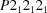

*a* = 9.847 (2) Å
*b* = 10.492 (3) Å
*c* = 17.819 (4) Å
*V* = 1841.0 (8) Å^3^
*Z* = 4
Cu *K*α radiation
μ = 2.92 mm^−1^
*T* = 296 K
0.20 × 0.19 × 0.18 mm

#### Data collection  

Bruker X8 Proteum diffractometer
Absorption correction: multi-scan (*SADABS*; Bruker, 2013 ▶ *T*_min_ = 0.593, *T*_max_ = 0.622
12103 measured reflections
3028 independent reflections
2547 reflections with *I* > 2σ(*I*)
*R*_int_ = 0.056

#### Refinement  

*R*[*F* ^2^ > 2σ(*F* ^2^)] = 0.085
*wR*(*F* ^2^) = 0.239
*S* = 1.03
3028 reflections
237 parameters
H-atom parameters constrained
Δρ_max_ = 0.94 e Å^−3^
Δρ_min_ = −0.51 e Å^−3^
Absolute structure: Flack (1983 ▶), 1270 Friedel pairs
Absolute structure parameter: 0.0 (3)

### 

Data collection: *APEX2* (Bruker, 2013 ▶); cell refinement: *SAINT* (Bruker, 2013 ▶); data reduction: *SAINT*; program(s) used to solve structure: *SHELXS97* (Sheldrick, 2008 ▶); program(s) used to refine structure: *SHELXL97* (Sheldrick, 2008 ▶); molecular graphics: *Mercury* (Macrae *et al.*, 2006 ▶); software used to prepare material for publication: *Mercury*.

## Supplementary Material

Crystal structure: contains datablock(s) global, I. DOI: 10.1107/S1600536814002529/jj2182sup1.cif

Structure factors: contains datablock(s) I. DOI: 10.1107/S1600536814002529/jj2182Isup2.hkl

Supporting information file. DOI: 10.1107/S1600536814002529/jj2182Isup3.cml

CCDC reference:

Additional supporting information:  crystallographic information; 3D view; checkCIF report

## Figures and Tables

**Table 1:** Hydrogen-bond geometry (Å, °)

*D*—H⋯*A*	*D*—H	H⋯*A*	*D*⋯*A*	*D*—H⋯*A*
C21—H21⋯N12^i^	0.93	2.52	3.262 (7)	137

Symmetry code: (i) 
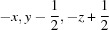
.

**Fig. 1.**
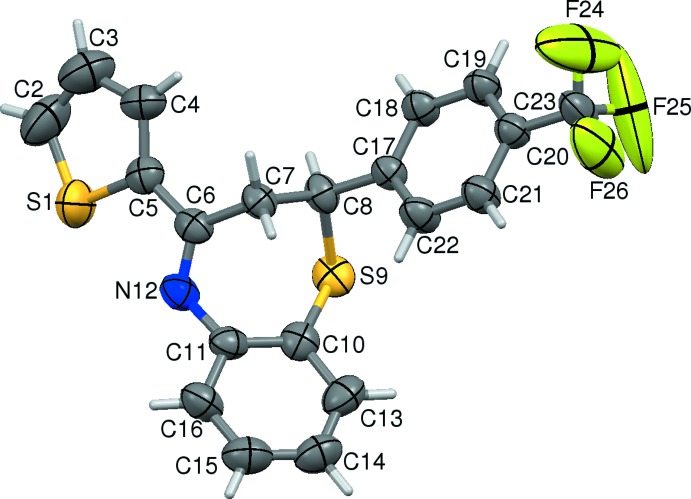
ORTEP diagram of the title molecule with 50% probability ellipsoids.

**Fig. 2.**
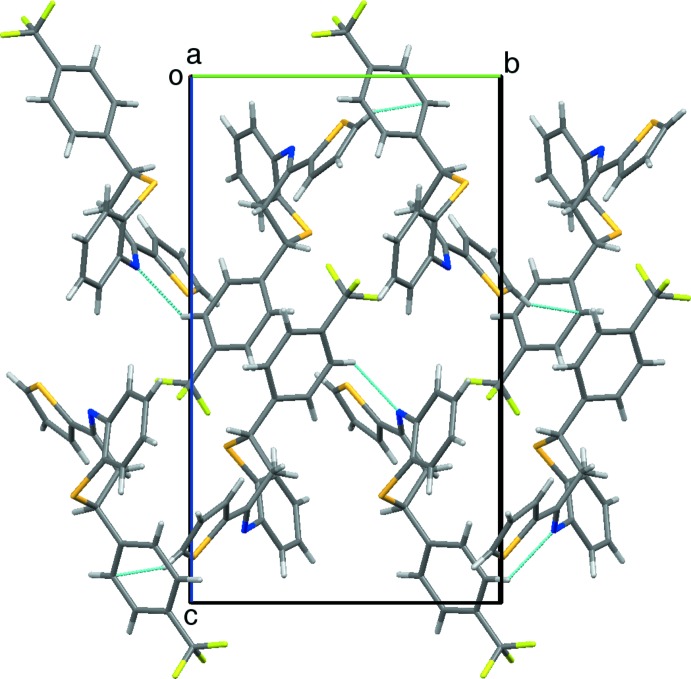
Packing diagram of molecule, viewed along the a axis. Dotted lines represent weak C—H···N intermolecular interactions.

## supplementary crystallographic information

### 1. Comment

1,4-Thiazepine is a privileged structure because of its presence in a number of pharmacologically important compounds. 1, 4-thiazepine moieties represent an important class of heterocyclic compounds with diverse potential and pharmacological activities. They are used as a calcium channel blockers, HIV-1 integrase and reverse transcriptase inhibitors, antitumor, antiplatelet and antidepressant agents (Zeng *et al.*, 2010). Compounds containing the 1, 4-thiazepine moiety are important targets in synthetic and medicinal chemistry because this fragment is a key motif in a wide number of natural and synthetic biologically active agents (Skiles *et al.*, 1986).

In the title compound, (I), the thiazepine ring adopts a slightly distorted twist-boat conformation. The mean plane of the five-membered thiophene ring fused to the thiazepine ring is twisted by 32.3 (3)° and 55.6 (4)° from the mean planes of the two benzene rings. In the crystal, the molecules are linked by weak C–H···N intermolecular interactions. The seven membered thiazepine ring adopts a twist boat conformation with the pairs of atoms N12/C11 and S9/C10 being oppositely oriented with respect to the C6/C7/C8 mean plane. The torsion angles around C6—C7 and C7—C8 are very close to the value of 52° reported for the corresponding torsion angle in the ideal twist boat conformation of cycloheptane. The bond lengths between C6—N12 is 1.29 (3)Å and C11—N12 is 1.39 (3)Å which is slightly less than the standard C—N value. The bond length between C8—S9 is 1.83 (9)Å and S9—C10 is 1.76 (3) Å. The sp2 and sp3 hybridization states of C10 and C8, respectively, account for the difference in the S—C bond lengths in (I). The bond lengths and angles do not show large deviations and are comparable with those reported for a similar structure (Manjula *et al.*, 2013).

### 2. Experimental

A mixture of (*Z*)-1-(thiophen-2-yl)-3-(4-(trifluoromethyl) phenyl)prop-2-en-1- one (3 mmol, 1 g) and 2-aminithiophenol (3 mmol, 0.4 g) with 3–4 drops of conc. HCl in methanol (10 ml) was heated with stirring at 433 K for 4 h. The reaction was monitored by thin-layer chromatography (hexane/ethyl acetate). After the completion of the reaction, the mixture was extracted in chloroform (30 ml), washed successively with dilute hydrochloric acid and water. The solvent was evaporated to dryness. The solid obtained was crystallized from 95° ethyl alcohol to get pale yellow needles of 4-(thiophen-2-yl)-2-(4- (trifluoromethyl) phenyl)-2,3-dihydrobenzo [*b*] [1,4] thiazepine in 80° yield.

### 3. Refinement

All hydrogen atoms were located geometrically with C—H = 0.93–0.97) Å and allowed to ride on their parent atoms with *U*_iso_(H) = 1.2*U*_eq_(aromatic C).

### Crystal data

**Table:**

C_20_H_14_F_3_NS_2_	*F*(000) = 800
*M**_r_* = 389.46	*D*_x_ = 1.405 Mg m^−^^3^
Orthorhombic, *P*2_1_2_1_2_1_	Cu *K*α radiation, λ = 1.54178 Å
Hall symbol: P 2ac 2ab	Cell parameters from 12103 reflections
*a* = 9.847 (2) Å	θ = 4.9–64.8°
*b* = 10.492 (3) Å	µ = 2.92 mm^−^^1^
*c* = 17.819 (4) Å	*T* = 296 K
*V* = 1841.0 (8) Å^3^	Needle, light yellow
*Z* = 4	0.20 × 0.19 × 0.18 mm

### Data collection

**Table:**

Bruker X8 Proteum diffractometer	3028 independent reflections
Radiation source: Bruker MicroStar microfocus rotating anode	2547 reflections with *I* > 2σ(*I*)
Helios multilayer optics monochromator	*R*_int_ = 0.056
Detector resolution: 10.7 pixels mm^-1^	θ_max_ = 64.8°, θ_min_ = 4.9°
φ and ω scans	*h* = −11→11
Absorption correction: multi-scan (*SADABS*; Bruker, 2013	*k* = −8→12
*T*_min_ = 0.593, *T*_max_ = 0.622	*l* = −20→20
12103 measured reflections	

### Refinement

**Table:**

Refinement on *F*^2^	Hydrogen site location: inferred from neighbouring sites
Least-squares matrix: full	H-atom parameters constrained
*R*[*F*^2^ > 2σ(*F*^2^)] = 0.085	*w* = 1/[σ^2^(*F*_o_^2^) + (0.1979*P*)^2^] where *P* = (*F*_o_^2^ + 2*F*_c_^2^)/3
*wR*(*F*^2^) = 0.239	(Δ/σ)_max_ < 0.001
*S* = 1.03	Δρ_max_ = 0.94 e Å^−^^3^
3028 reflections	Δρ_min_ = −0.51 e Å^−^^3^
237 parameters	Extinction correction: *SHELXL*, FC^*^=KFC[1+0.001XFC^2^Λ^3^/SIN(2Θ)]^-1/4^
0 restraints	Extinction coefficient: 0.017 (3)
Primary atom site location: structure-invariant direct methods	Absolute structure: Flack (1983), 1270 Friedel pairs
Secondary atom site location: difference Fourier map	Absolute structure parameter: 0.0 (3)

### Special details

**Table:**

Experimental. m.p. 405 K, 1H NMR (CDCl3): δ 2.0 (d, 2H, C3—H), 3.80 (t, 1H, C2—H), 7.24 (dd, 2H, Ar—H), 7.58 (dd, 2H, Ar—H), 7.18 (t, 1H, C4—H 5 m ring), 7.46 (d, 1H, C3—H 5 m ring), 7.72 (d, 1H, C5—H 5 m ring), 7.32–7.46 (m, 4H, Ar—H). Anal. Calcd. for C20H14F3NS2: C 62.14, H 3.56, N 3.58°; Found C 61.68, H 3.62, N 3.60°. Mass FAB+ (NBA): 390 (*M* + 1, 100°).
Geometry. Bond distances, angles *etc*. have been calculated using the rounded fractional coordinates. All su's are estimated from the variances of the (full) variance-covariance matrix. The cell e.s.d.'s are taken into account in the estimation of distances, angles and torsion angles
Refinement. Refinement on *F*^2^ for ALL reflections except those flagged by the user for potential systematic errors. Weighted *R*-factors *wR* and all goodnesses of fit *S* are based on *F*^2^, conventional *R*-factors *R* are based on *F*, with *F* set to zero for negative *F*^2^. The observed criterion of *F*^2^ > σ(*F*^2^) is used only for calculating -*R*-factor-obs *etc*. and is not relevant to the choice of reflections for refinement. *R*-factors based on *F*^2^ are statistically about twice as large as those based on *F*, and *R*-factors based on ALL data will be even larger.

### Fractional atomic coordinates and isotropic or equivalent isotropic displacement parameters (Å^2^)

**Table:**

	*x*	*y*	*z*	*U*_iso_*/*U*_eq_	
S1	0.3240 (2)	0.99443 (19)	0.41340 (10)	0.0841 (7)	
S9	−0.00510 (15)	0.88544 (12)	0.20297 (9)	0.0608 (5)	
F24	0.2469 (9)	0.4640 (10)	−0.1093 (6)	0.257 (6)	
F25	0.0847 (19)	0.5559 (8)	−0.1388 (4)	0.289 (10)	
F26	0.0674 (7)	0.3951 (6)	−0.0787 (3)	0.139 (3)	
N12	0.1053 (5)	0.8288 (4)	0.3632 (3)	0.0550 (16)	
C2	0.4905 (8)	1.0319 (9)	0.4023 (6)	0.104 (4)	
C3	0.5488 (8)	0.9699 (9)	0.3449 (7)	0.101 (4)	
C4	0.4592 (6)	0.8887 (6)	0.3089 (4)	0.0650 (19)	
C5	0.3308 (5)	0.8882 (5)	0.3403 (3)	0.0533 (17)	
C6	0.2105 (5)	0.8181 (5)	0.3202 (3)	0.0500 (16)	
C7	0.2105 (5)	0.7370 (5)	0.2509 (3)	0.0477 (16)	
C8	0.1595 (5)	0.8108 (4)	0.1818 (3)	0.0510 (16)	
C10	−0.0758 (6)	0.7848 (5)	0.2719 (3)	0.0523 (16)	
C11	−0.0168 (5)	0.7715 (5)	0.3428 (3)	0.0517 (14)	
C13	−0.2004 (6)	0.7269 (6)	0.2580 (4)	0.0680 (19)	
C14	−0.2705 (6)	0.6610 (6)	0.3132 (5)	0.071 (2)	
C15	−0.2116 (7)	0.6511 (6)	0.3834 (5)	0.073 (2)	
C16	−0.0877 (7)	0.7058 (6)	0.3996 (4)	0.0653 (19)	
C17	0.1511 (5)	0.7293 (4)	0.1124 (3)	0.0447 (12)	
C18	0.2330 (6)	0.7568 (5)	0.0509 (3)	0.0503 (16)	
C19	0.2279 (6)	0.6822 (5)	−0.0137 (3)	0.0557 (17)	
C20	0.1419 (5)	0.5784 (5)	−0.0162 (3)	0.0493 (16)	
C21	0.0616 (6)	0.5477 (5)	0.0452 (3)	0.0580 (17)	
C22	0.0667 (6)	0.6223 (5)	0.1088 (3)	0.0580 (17)	
C23	0.1334 (7)	0.4983 (7)	−0.0842 (3)	0.071 (2)	
H2	0.53640	1.08950	0.43280	0.1250*	
H3	0.63920	0.98020	0.33100	0.1210*	
H4	0.48310	0.83940	0.26760	0.0780*	
H7A	0.15280	0.66340	0.25910	0.0570*	
H7B	0.30190	0.70660	0.24150	0.0570*	
H8	0.22470	0.87940	0.17180	0.0610*	
H13	−0.23780	0.73260	0.21020	0.0820*	
H14	−0.35470	0.62440	0.30330	0.0850*	
H15	−0.25690	0.60620	0.42080	0.0880*	
H16	−0.05120	0.69950	0.44760	0.0790*	
H18	0.29200	0.82590	0.05300	0.0600*	
H19	0.28190	0.70230	−0.05490	0.0660*	
H21	0.00450	0.47720	0.04340	0.0700*	
H22	0.01320	0.60120	0.14990	0.0700*	

### Atomic displacement parameters (Å^2^)

**Table:**

	*U*^11^	*U*^22^	*U*^33^	*U*^12^	*U*^13^	*U*^23^
S1	0.0843 (11)	0.0942 (13)	0.0738 (11)	−0.0174 (9)	−0.0048 (8)	−0.0297 (9)
S9	0.0687 (9)	0.0503 (7)	0.0634 (9)	0.0168 (6)	0.0018 (7)	0.0040 (5)
F24	0.190 (8)	0.307 (12)	0.275 (12)	−0.134 (8)	0.144 (8)	−0.238 (11)
F25	0.64 (3)	0.152 (6)	0.076 (4)	0.034 (12)	−0.127 (9)	−0.021 (4)
F26	0.200 (7)	0.123 (4)	0.093 (4)	−0.086 (4)	0.037 (4)	−0.050 (3)
N12	0.063 (3)	0.050 (2)	0.052 (3)	−0.002 (2)	0.010 (2)	−0.0088 (18)
C2	0.073 (4)	0.106 (6)	0.133 (8)	−0.017 (4)	−0.026 (5)	−0.019 (6)
C3	0.067 (4)	0.104 (6)	0.132 (8)	−0.018 (4)	0.003 (5)	−0.001 (6)
C4	0.051 (3)	0.070 (3)	0.074 (4)	−0.003 (2)	0.011 (3)	0.004 (3)
C5	0.053 (3)	0.050 (3)	0.057 (3)	0.004 (2)	−0.001 (2)	0.003 (2)
C6	0.054 (3)	0.046 (2)	0.050 (3)	0.008 (2)	0.005 (2)	0.002 (2)
C7	0.055 (3)	0.042 (2)	0.046 (3)	0.004 (2)	0.004 (2)	−0.005 (2)
C8	0.064 (3)	0.045 (2)	0.044 (3)	0.004 (2)	0.001 (2)	−0.0035 (19)
C10	0.057 (3)	0.040 (2)	0.060 (3)	0.008 (2)	0.000 (2)	−0.005 (2)
C11	0.050 (2)	0.050 (2)	0.055 (3)	−0.002 (2)	0.012 (2)	−0.010 (2)
C13	0.049 (3)	0.067 (3)	0.088 (4)	0.005 (3)	−0.008 (3)	−0.008 (3)
C14	0.054 (3)	0.064 (3)	0.095 (5)	−0.010 (3)	0.002 (3)	−0.002 (3)
C15	0.063 (3)	0.067 (4)	0.089 (5)	−0.005 (3)	0.017 (4)	0.003 (3)
C16	0.075 (4)	0.064 (3)	0.057 (3)	−0.007 (3)	0.018 (3)	−0.001 (3)
C17	0.052 (2)	0.039 (2)	0.043 (2)	−0.0020 (19)	0.0043 (19)	0.0005 (19)
C18	0.057 (3)	0.045 (2)	0.049 (3)	−0.007 (2)	0.004 (2)	0.005 (2)
C19	0.061 (3)	0.057 (3)	0.049 (3)	−0.014 (2)	0.008 (2)	0.000 (2)
C20	0.053 (3)	0.049 (2)	0.046 (3)	−0.004 (2)	0.005 (2)	0.000 (2)
C21	0.066 (3)	0.055 (3)	0.053 (3)	−0.009 (2)	0.010 (3)	−0.003 (2)
C22	0.067 (3)	0.053 (3)	0.054 (3)	−0.016 (2)	0.014 (2)	−0.005 (2)
C23	0.087 (4)	0.080 (4)	0.047 (3)	−0.026 (4)	0.018 (3)	−0.012 (3)

### Geometric parameters (Å, º)

**Table:**

S1—C2	1.698 (8)	C17—C18	1.391 (8)
S1—C5	1.716 (6)	C17—C22	1.398 (7)
S9—C8	1.839 (5)	C18—C19	1.393 (8)
S9—C10	1.763 (6)	C19—C20	1.380 (8)
F24—C23	1.256 (11)	C20—C21	1.388 (8)
F25—C23	1.242 (12)	C20—C23	1.477 (8)
F26—C23	1.267 (10)	C21—C22	1.378 (8)
N12—C6	1.293 (7)	C2—H2	0.9300
N12—C11	1.393 (7)	C3—H3	0.9300
C2—C3	1.341 (15)	C4—H4	0.9300
C3—C4	1.384 (12)	C7—H7A	0.9700
C4—C5	1.383 (8)	C7—H7B	0.9700
C5—C6	1.440 (7)	C8—H8	0.9800
C6—C7	1.500 (8)	C13—H13	0.9300
C7—C8	1.539 (7)	C14—H14	0.9300
C8—C17	1.506 (7)	C15—H15	0.9300
C10—C11	1.398 (8)	C16—H16	0.9300
C10—C13	1.391 (8)	C18—H18	0.9300
C11—C16	1.410 (9)	C19—H19	0.9300
C13—C14	1.386 (10)	C21—H21	0.9300
C14—C15	1.383 (12)	C22—H22	0.9300
C15—C16	1.379 (10)		
			
C2—S1—C5	91.4 (4)	F24—C23—F25	101.8 (10)
C8—S9—C10	103.6 (2)	F24—C23—F26	103.8 (8)
C6—N12—C11	120.0 (5)	F24—C23—C20	113.9 (7)
S1—C2—C3	113.0 (7)	F25—C23—F26	106.2 (8)
C2—C3—C4	112.3 (7)	F25—C23—C20	112.8 (7)
C3—C4—C5	113.4 (7)	F26—C23—C20	116.9 (5)
S1—C5—C4	109.9 (4)	S1—C2—H2	123.00
S1—C5—C6	119.3 (4)	C3—C2—H2	124.00
C4—C5—C6	130.8 (5)	C2—C3—H3	124.00
N12—C6—C5	117.9 (5)	C4—C3—H3	124.00
N12—C6—C7	122.5 (5)	C3—C4—H4	123.00
C5—C6—C7	119.7 (4)	C5—C4—H4	123.00
C6—C7—C8	111.9 (4)	C6—C7—H7A	109.00
S9—C8—C7	109.7 (4)	C6—C7—H7B	109.00
S9—C8—C17	111.2 (3)	C8—C7—H7A	109.00
C7—C8—C17	112.9 (4)	C8—C7—H7B	109.00
S9—C10—C11	121.7 (4)	H7A—C7—H7B	108.00
S9—C10—C13	119.1 (5)	S9—C8—H8	108.00
C11—C10—C13	118.9 (5)	C7—C8—H8	108.00
N12—C11—C10	123.5 (5)	C17—C8—H8	108.00
N12—C11—C16	116.8 (5)	C10—C13—H13	119.00
C10—C11—C16	119.5 (5)	C14—C13—H13	119.00
C10—C13—C14	122.0 (6)	C13—C14—H14	121.00
C13—C14—C15	118.1 (6)	C15—C14—H14	121.00
C14—C15—C16	122.0 (7)	C14—C15—H15	119.00
C11—C16—C15	119.4 (7)	C16—C15—H15	119.00
C8—C17—C18	119.8 (4)	C11—C16—H16	120.00
C8—C17—C22	121.8 (5)	C15—C16—H16	120.00
C18—C17—C22	118.4 (5)	C17—C18—H18	119.00
C17—C18—C19	120.9 (5)	C19—C18—H18	120.00
C18—C19—C20	119.5 (5)	C18—C19—H19	120.00
C19—C20—C21	120.5 (5)	C20—C19—H19	120.00
C19—C20—C23	120.7 (5)	C20—C21—H21	120.00
C21—C20—C23	118.8 (5)	C22—C21—H21	120.00
C20—C21—C22	119.7 (5)	C17—C22—H22	119.00
C17—C22—C21	121.0 (5)	C21—C22—H22	119.00
			
C5—S1—C2—C3	1.5 (8)	S9—C10—C11—C16	170.3 (4)
C2—S1—C5—C4	−1.9 (5)	C13—C10—C11—N12	−177.9 (5)
C2—S1—C5—C6	179.5 (5)	S9—C10—C13—C14	−171.0 (5)
C10—S9—C8—C7	−27.7 (4)	C11—C10—C13—C14	2.8 (9)
C10—S9—C8—C17	97.9 (4)	C13—C10—C11—C16	−3.3 (8)
C8—S9—C10—C11	65.2 (5)	S9—C10—C11—N12	−4.3 (8)
C8—S9—C10—C13	−121.2 (5)	N12—C11—C16—C15	177.7 (5)
C6—N12—C11—C16	135.8 (6)	C10—C11—C16—C15	2.7 (9)
C11—N12—C6—C5	174.6 (5)	C10—C13—C14—C15	−1.5 (10)
C11—N12—C6—C7	−4.9 (8)	C13—C14—C15—C16	0.8 (10)
C6—N12—C11—C10	−49.5 (7)	C14—C15—C16—C11	−1.5 (10)
S1—C2—C3—C4	−0.7 (11)	C8—C17—C18—C19	179.5 (5)
C2—C3—C4—C5	−0.7 (11)	C22—C17—C18—C19	2.1 (8)
C3—C4—C5—S1	1.8 (8)	C8—C17—C22—C21	−179.1 (5)
C3—C4—C5—C6	−179.8 (7)	C18—C17—C22—C21	−1.8 (8)
S1—C5—C6—N12	−6.9 (7)	C17—C18—C19—C20	−1.0 (8)
C4—C5—C6—N12	174.9 (6)	C18—C19—C20—C21	−0.4 (8)
C4—C5—C6—C7	−5.6 (9)	C18—C19—C20—C23	179.4 (5)
S1—C5—C6—C7	172.7 (4)	C19—C20—C21—C22	0.7 (8)
N12—C6—C7—C8	87.7 (6)	C23—C20—C21—C22	−179.1 (5)
C5—C6—C7—C8	−91.8 (6)	C19—C20—C23—F24	49.1 (9)
C6—C7—C8—C17	−176.8 (4)	C19—C20—C23—F25	−66.2 (11)
C6—C7—C8—S9	−52.1 (5)	C19—C20—C23—F26	170.3 (6)
C7—C8—C17—C18	−116.1 (5)	C21—C20—C23—F24	−131.0 (8)
S9—C8—C17—C18	120.0 (5)	C21—C20—C23—F25	113.6 (11)
S9—C8—C17—C22	−62.8 (5)	C21—C20—C23—F26	−9.9 (9)
C7—C8—C17—C22	61.1 (6)	C20—C21—C22—C17	0.4 (8)

### Hydrogen-bond geometry (Å, º)

**Table:**

*D*—H···*A*	*D*—H	H···*A*	*D*···*A*	*D*—H···*A*
C21—H21···N12^i^	0.93	2.52	3.262 (7)	137

Symmetry code: (i) −*x*, *y*−1/2, −*z*+1/2.
